# Supplementary material for: Physiological thermal responses of three Mexican snakes with distinct lifestyles
Source: PeerJ. 2024 Jul 19;12:e17705. doi: 10.7717/peerj.17705 (PMC11262299; doi:10.7717/peerj.17705)
Supplement: Supplemental Information 2 — Example of the calculations made to get swimming speed from an individual of Crotalus polystictus. Maximum speed is marked in bold. [file peerj-12-17705-s002.docx]

**Supplemental material**

**Table S1.** Example of the calculations made to estimate swimming speed from an individual of *Crotalus polystictus*. Maximum speed is marked in bold.

| **Snake ID** | **Treatment (°C)** | **Distance (cm)** | **Time mark** | **Frame count**  **(60 fps)** | **Time (s)** | **Speed (cm/s)** |
| --- | --- | --- | --- | --- | --- | --- |
| *Crotalus polystictus* #25 | 15 | 0 | 10.18 | - | - | - |
|  |  | 30 | 12.07 | 109 | 1.81666667 | 16.5137615 |
|  |  | 0 | 21.38 | - | - | - |
|  |  | 30 | 23.07 | 89 | 1.48333333 | 20.2247191 |
|  |  | 0 | 54.16 | - | - | - |
|  |  | 30 | 56.01 | 105 | 1.75 | 17.1428571 |
|  |  | 60 | 57.19 | 78 | 1.3 | 23.0769231 |
|  |  | 90 | 58.33 | 74 | 1.23333333 | **24.3243243** |
|  |  | 120 | 1.00.23 | 110 | 1.83333333 | 16.3636364 |
|  |  | 150 | 1.01.48 | 85 | 1.41666667 | 21.1764706 |
|  |  | 180 | 1.03.49 | 121 | 2.01666667 | 14.8760331 |
|  |  | 210 | 1.05.46 | 117 | 1.95 | 15.3846154 |
|  |  | 240 | 1.07.46 | 120 | 2 | 15 |
| *Crotalus polystictus* #25 | 25 | 0 | 9.35 | - | - | - |
|  |  | 30 | 10.51 | 76 | 1.26666667 | 23.6842105 |
|  |  | 60 | 11.41 | 50 | 0.83333333 | 36 |
|  |  | 90 | 12.18 | 37 | 0.61666667 | 48.6486486 |
|  |  | 120 | 12.57 | 39 | 0.65 | 46.1538462 |
|  |  | 150 | 13.32 | 35 | 0.58333333 | **51.4285714** |
|  |  | 180 | 14.29 | 57 | 0.95 | 31.5789474 |
|  |  | 0 | 16.25 | - | - | - |
|  |  | 30 | 17.08 | 43 | 0.71666667 | 41.8604651 |
|  |  | 60 | 17.57 | 49 | 0.81666667 | 36.7346939 |
| *Crotalus polystictus* #25 | 30 | 0 | 12.27 | - | - | - |
|  |  | 30 | 13.46 | 81 | 1.35 | 22.2222222 |
|  |  | 60 | 14.31 | 45 | 0.75 | 40 |
|  |  | 0 | 17.53 | - | - | - |
|  |  | 30 | 18.39 | 46 | 0.76666667 | 39.1304348 |
|  |  | 60 | 19.56 | 78 | 1.3 | 23.0769231 |
|  |  | 90 | 20.58 | 62 | 1.03333333 | 29.0322581 |
|  |  | 0 | 27.17 | - | - | - |
|  |  | 30 | 28.06 | 49 | 0.81666667 | 36.7346939 |
|  |  | 60 | 29.03 | 57 | 0.95 | 31.5789474 |
|  |  | 90 | 29.42 | 39 | 0.65 | 46.1538462 |
|  |  | 120 | 30.19 | 37 | 0.61666667 | **48.6486486** |
|  |  | 150 | 31.01 | 42 | 0.7 | 42.8571428 |

**Table S1. Continuation.** Example of the calculations made to get swimming speed from an individual of *Crotalus polystictus*. Maximum speed is marked in bold.

| **Snake ID** | **Treatment (°C)** | **Distance (cm)** | **Time mark** | **Frame count (60 fps)** | **Time (s)** | **Speed (cm/s)** |
| --- | --- | --- | --- | --- | --- | --- |
| *Crotalus polystictus* #25 | 33 | 0 | 14.35 | - | - | - |
|  |  | 30 | 15.28 | 53 | 0.88333333 | 33.9622642 |
|  |  | 60 | 16.18 | 50 | 0.83333333 | 36 |
|  |  | 90 | 17.15 | 57 | 0.95 | 31.5789474 |
|  |  | 120 | 17.56 | 41 | 0.68333333 | 43.902439 |
|  |  | 150 | 18.35 | 39 | 0.65 | 46.1538462 |
|  |  | 180 | 19.12 | 37 | 0.61666667 | **48.6486486** |
|  |  | 210 | 19.53 | 41 | 0.68333333 | 43.902439 |
|  |  | 0 | 38.50 | - | - | - |
|  |  | 30 | 39.30 | 40 | 0.66666667 | 45 |
|  |  | 60 | 40.50 | 80 | 1.33333333 | 22.5 |
|  |  | 90 | 42.14 | 84 | 1.4 | 21.4285714 |
|  |  | 120 | 43.50 | 96 | 1.6 | 18.75 |
|  |  | 150 | 44.44 | 54 | 0.9 | 33.3333333 |
|  |  | 180 | 45.23 | 39 | 0.65 | 46.1538462 |
| *Crotalus polystictus* #25 | 36 | 0 | 4.42 | - | - | - |
|  |  | 30 | 5.47 | 65 | 1.08333333 | 27.6923077 |
|  |  | 60 | 6.26 | 39 | 0.65 | 46.1538462 |
|  |  | 90 | 7.01 | 35 | 0.58333333 | 51.4285714 |
|  |  | 120 | 7.33 | 32 | 0.53333333 | **56.25** |
|  |  | 150 | 8.06 | 33 | 0.55 | 54.5454545 |
|  |  | 180 | 8.39 | 33 | 0.55 | 54.5454545 |
|  |  | 210 | 9.14 | 35 | 0.58333333 | 51.4285714 |
|  |  | 240 | 9.54 | 40 | 0.66666667 | 45 |
|  |  | 270 | 10.32 | 38 | 0.63333333 | 47.3684211 |
