# Supplementary material for: Physiological thermal responses of three Mexican snakes with distinct lifestyles
Source: PeerJ. 2024 Jul 19;12:e17705. doi: 10.7717/peerj.17705 (PMC11262299; doi:10.7717/peerj.17705)
Supplement: Supplemental Information 3 — TPCs fitted for the study species with the highest R2. The best curve (in bold) was selected by the lowest AICc. [file peerj-12-17705-s003.docx]

**Table S2.** TPCs fitted for the study species with the highest R^2^. The best curve (in bold) was selected by the lowest AICc.

| *Crotalus polystictus* | | | |
| --- | --- | --- | --- |
| Function | AICc | Delta AICc | R^2^ |
| **Exponentially modified Gaussian** | **-98.172** | **0** | **0.91694** |
| GMG | -95.648 | 2.524 | 0.95024 |
| Beta | -56.396 | 41.776 | 0.90713 |
| Pearson IV | -56.306 | 41.866 | 0.9112 |
|  |  |  |  |
| *Conopsis lineata* | | | |
| Function | AICc | Delta AICc | R^2^ |
| **Asymmetric logistic** | **-94.907** | **0** | **0.94230** |
| Extreme Value 4-P Fronted | -94.833 | 3.339 | 0.94563 |
| Pearson IV | -53.118 | 45.054 | 0.93731 |
| Asymmetric double sigmoidal | -47.534 | 50.638 | 0.99616 |
| Asymmetric double Gaussian | -47.470 | 50.702 | 0.99628 |
|  |  |  |  |
| *Thamnophis melanogaster* | | | |
| Function | AICc | Delta AICc | R^2^ |
| **Extreme Value 4-P Fronted** | **-99.016** | **0** | **0.92839** |
| Exponentially modified Gaussian | -98.485 | 0.531 | 0.94509 |
| Log Normal-4P | -98.420 | 0.596 | 0.94684 |
| Pearson IV | -56.760 | 42.256 | 0.93700 |
| Asymmetric double sigmoidal | -53.118 | 45.898 | 0.99826 |
