# Supplementary material for: Physiological thermal responses of three Mexican snakes with distinct lifestyles
Source: PeerJ. 2024 Jul 19;12:e17705. doi: 10.7717/peerj.17705 (PMC11262299; doi:10.7717/peerj.17705)
Supplement: Supplemental Information 4 [file peerj-12-17705-s004.docx]

**Table S3**. Model selection results for analyses of RMR in the study species.

| **Models** | **K** | **AICc** | **Delta AICc** |
| --- | --- | --- | --- |
| ***Crotalus polystictus*** |  |  |  |
| Temperature + Body mass | 8 | -240.681 | 0 |
| Temperature*Body mass | 12 | -232.596 | 8.085 |
| Temperature + Body mass + Sex | 10 | -227.470 | 13.211 |
| Temperature*Body mass + Sex | 14 | -219.271 | 21.410 |
| Temperature | 7 | -120.358 | 120.323 |
| Body mass | 4 | -83.868 | 156.813 |
| Body mass + Sex | 6 | -70.836 | 169.845 |
| Null | 3 | 26.036 | 266.717 |
| ***Conopsis lineata*** |  |  |  |
| Temperature + Body mass | 8 | -65.720 | 0 |
| Temperature*Body mass | 12 | -56.363 | 9.356 |
| Temperature + Body mass + Sex | 10 | -55.984 | 9.736 |
| Temperature | 7 | -49.138 | 16.582 |
| Temperature*Body mass + Sex | 14 | -46.058 | 19.662 |
| Body mass | 4 | 55.248 | 120.968 |
| Body mass + Sex | 6 | 64.956 | 130.676 |
| Null | 3 | 69.163 | 134.883 |
| ***Thamnophis melanogaster*** |  |  |  |
| Temperature + Body mass | 8 | -115.993 | 0 |
| Temperature*Body mass | 12 | -109.972 | 6.021 |
| Temperature + Body mass + Sex | 10 | -103.761 | 12.232 |
| Temperature*Body mass + Sex | 14 | -97.360 | 18.633 |
| Temperature | 7 | -26.440 | 89.553 |
| Body mass | 4 | 92.394 | 208.387 |
| Body mass + Sex | 6 | 102.913 | 218.906 |
| Null | 3 | 165.493 | 281.485 |
