# Supplementary material for: Physiological thermal responses of three Mexican snakes with distinct lifestyles
Source: PeerJ. 2024 Jul 19;12:e17705. doi: 10.7717/peerj.17705 (PMC11262299; doi:10.7717/peerj.17705)
Supplement: Supplemental Information 5 — Mean values of RMR ± SE per thermal treatment for the study species. [file peerj-12-17705-s005.docx]

**Table S4.** Mean values of RMR ± SE per thermal treatment for the study species.

| Species | Thermal treatment (°C) | RMR  (CO_2_ ml/min) |
| --- | --- | --- |
| *Crotalus polystictus* | 15 | 3.582 ± 0.443 |
|  | 25 | 5.114 ± 0.546 |
|  | 30 | 5.898 ± 0.665 |
|  | 33 | 6.495 ± 1.084 |
|  | 36 | 10.086 ± 1.119 |
| *Conopsis lineata* | 15 | 0.344 ± 0.040 |
|  | 25 | 0.813 ± 0.077 |
|  | 30 | 1.106 ± 0.097 |
|  | 33 | 1.250 ± 0.093 |
|  | 36 | 1.807 ± 0.201 |
| *Thamnophis melanogaster* | 15 | 1.056 ± 0.172 |
|  | 25 | 2.355 ± 0.360 |
|  | 30 | 3.179 ± 0.383 |
|  | 33 | 3.320 ± 0.448 |
|  | 36 | 5.067 ± 0.598 |
